# Envelope Glycoprotein of Arenaviruses

**DOI:** 10.3390/v4102162

**Published:** 2012-10-17

**Authors:** Dominique J. Burri, Joel Ramos da Palma, Stefan Kunz, Antonella Pasquato

**Affiliations:** Institute of Microbiology, University Hospital Center and University of Lausanne, Lausanne, Rue du Bugnon 48, 1011, Switzerland; Email: dominique.burri@chuv.ch (DJ.B); joel.palma@chuv.ch (J.R.P); stefan.kunz@chuv.ch (S.K)

**Keywords:** Arenavirus, anti-viral, drug, GPC, SKI-1/S1P, PF-429242

## Abstract

Arenaviruses include lethal human pathogens which pose serious public health threats. So far, no FDA approved vaccines are available against arenavirus infections, and therapeutic options are limited, making the identification of novel drug targets for the development of efficacious therapeutics an urgent need. Arenaviruses are comprised of two RNA genome segments and four proteins, the polymerase L, the envelope glycoprotein GP, the matrix protein Z, and the nucleoprotein NP. A crucial step in the arenavirus life-cycle is the biosynthesis and maturation of the GP precursor (GPC) by cellular signal peptidases and the cellular enzyme Subtilisin Kexin Isozyme-1 (SKI-1)/Site-1 Protease (S1P) yielding a tripartite mature GP complex formed by GP1/GP2 and a stable signal peptide (SSP). GPC cleavage by SKI-1/S1P is crucial for fusion competence and incorporation of mature GP into nascent budding virion particles. In a first part of our review, we cover basic aspects and newer developments in the biosynthesis of arenavirus GP and its molecular interaction with SKI-1/S1P. A second part will then highlight the potential of SKI-1/S1P-mediated processing of arenavirus GPC as a novel target for therapeutic intervention to combat human pathogenic arenaviruses.

## 1. Introduction

### 1.1. Arenaviruses are important human pathogens

Arenaviruses are world-wide distributed negative strand RNA viruses that include a number of important human pathogens. The prototypic arenavirus lymphocytic choriomeningitis virus (LCMV) was discovered by Armstrong and Lille upon passage of infectious material derived from a fatal case of encephalitis during the 1933 outbreak in St Louis (USA) [1]. Independently and almost at the same time, Rivers and Scott were able to isolate the same virus from patients with manifestations of aseptic meningitis [2], while Traub established that asymptomatic healthy mice can be carriers of the virus isolated from a mouse colony at the Rockefeller Institute [3]. During the following 30 years, several other viruses sharing the same unique features of LCMV were discovered and described giving rise to a new family of virus named *Arenaviridae*. The “sandy” appearance of the viral particles in electron microscopy, due to the presence of host ribosomes, is at the origin of the family name, which has its roots in the Latin word “*arena”*, meaning sand.

Currently, 22 species of arenaviruses, belonging to one singe genus, are recognized [4]. Based on serological, genetic and geographical data, arenaviruses are divided into two major subgroups: the Old World (OW) and the New World (NW) complex. The Old World lineage consists of the prototypic LCMV and other viruses endemic to the African continent, including Lassa (LASV), Mopeia (MOPV), Ippy, and Mobala (MOBV) viruses. The larger New World complex is further divided into three clades, A, B and C. Clade B is the most important in term of human disease, since it contains the major viruses causing hemorrhagic fevers (HF) in South America, i.e. Junín (JUNV), Machupo (MACV), Guanarito (GTOV) and Sabia (SABV) viruses but also other non-pathogenic viruses, like Tacaribe (TCRV) and Amapari virus (AMPV). The geographical distribution of the different viral species is intrinsically connected to the distribution of their natural hosts, and inherently the specific diseases associated to each virus follow a similar spatial distribution.

Several arenaviruses are causative agents of severe viral hemorrhagic fevers (VHF) in humans which are among the most devastating human diseases [5]. Among these, LASV is a major concern due to its high prevalence in Western Africa, where it causes several hundred thousand cases of Lassa fever every year, associated with high levels of morbidity and mortality [6]. Likewise, VHFs are also associated with several arenaviruses endemic in the South American continent, including JUNV, MACV, GTOV, and SABV, which are etiological agents of severe VHF in Argentina, Venezuela, Bolivia, and Brazil, respectively [7,8]. Among these, JUNV raises most concern due to its seasonal epidemics of Argentine hemorrhagic fevers [9,10] and high prevalence in rural areas while MACV, GTOV and SABV cause only sporadic outbreaks. The viral hemorrhagic fevers caused by arenaviruses are characterized by a broad range of clinical signs and symptoms and the differential diagnosis based on early clinical symptoms is often difficult. After and incubation period of 1-2 weeks, arenavirus VHF patients develop fever, weakness and general malaise, including cough, severe headache, and sore throat. Gastrointestinal symptoms are usually present with nausea, diarrhea, and vomiting. Clinical complications such as pleural effusions, facial edema, neurological complications and bleeding from mucosal surfaces indicate a poor diagnosis. In severe cases, deterioration is rapid and at terminal stage patients often succumb to shock, although the mild blood loss alone does not account for the death of the patient. 

The worldwide distributed LCMV is recognized as a neglected human pathogen of clinical significance. Being rarely fatal in immune-competent individuals causing only asymptomatic or mild, self-limited disease, it represents however a major risk to transplanted patients under immune-suppressive treatment [11]. LCMV further poses a significant risk to the fetus and the newborn *via* vertical transmission from acutely infected mothers *in utero* or perinatally resulting in severe brain malformation including hydrocephalus, chorioretinitis and mental retardation [12]. Employees of rodent breeding facilities are also at risk due to the exposure to chronicle infected animals as illustrated by a recent study conducted by the CDC where a mice breeding facility in Indiana (USA) showed that 20% of the mice had LCMV-reactive serum and 25% of the facility workers showed immunological evidence of recent LCMV exposure [13].

Of concern is also the fast emergence of new arenavirus associated with VHF syndromes. Recent examples are Chapare virus, discovered in Bolivia, which is closely related to other South American arenaviruses [14], and Lujo virus, identified during a nosocomial outbreak of VHF with high mortality in Zambia and South Africa in 2008 that caused the death of four out of five patients [15]. The discovery of this virus represents the first new HF-associated arenavirus to be isolated and identified in Africa in nearly 40 years. The continued emergence of previously unrecognized arenaviruses associated with fatal human disease in recent years suggests that many others could be identified in the future, raising serious concerns. Furthermore, increasing international air traffic coming from regions where arenavirus VHFs are endemic raises the fear of importation into non-endemic regions, putting local populations at risk. Indeed, since 1970, there have been several cases of Lassa fever imported into Europe or North America [16]. 

### 1.2. Arenavirus life-cycle

**Figure 1:**
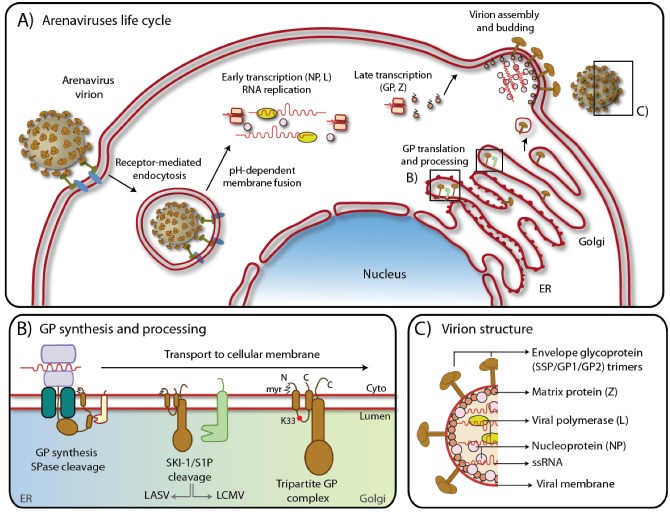
**A**) Schematic representation of arenavirus life cycle. Boxed regions refer to **B**) and **C**). For details, please see text. **B**) Biosynthesis of GPC in the ER with SSP cleavage by signal peptidase and maturation by the cellular protease SKI-1/S1P along the secretory pathway. The myristoylation modification and lysine 33 are also shown. Note that while LASV GP is cleaved early in the ER, LCMV GP is cleaved later in Golgi/TGN [38]. C) Arenavirus virion structure showing the tripartite GP protein complex, the Zinc finger matrix protein (Z), the RNA dependent polymerase (L), the nucleoprotein (NP) and the negative single stranded ambisense RNA genome.

Arenavirus replication has been covered by excellent recent reviews [17,18] and only a short overview will be given here. All arenaviruses consist of a nucleocapsid surrounded by a membrane envelope and have a non-lytic life cycle restricted to the cytoplasm (Figure 1A). Arenaviruses use an ambisense coding strategy and each genomic segment, L and S directs the synthesis of two polypeptides in opposite orientations, separated by a noncoding intergenic region (IGR) [19]. The S RNA encodes the nucleoprotein (NP; ca 63 kDa) and the viral glycoprotein precursor (GPC; ca 75 kDa) that is post-translationally cleaved by a cellular enzyme into the mature virion glycoproteins (GPs) GP1(44 kDa) and GP2 (35 kDa) (Figure 1B). Mature GPs form trimers [20]. The L RNA encodes the viral RNA-dependent RNA polymerase (RdRp, or L polymerase; ca 200 kDa), and a small RING finger protein (Z; ca 11 kDa) [21] (Figure 1C). This ambisense organization means that while the NP and L proteins are translated directly from the genomic complementary mRNA, the GPC and Z proteins are synthesized from the genomic sense mRNAs that are transcribed using the corresponding antigenome RNA (agRNA) species [21]. Since the genomic RNA of arenaviruses cannot serve directly as a template for translation, arenaviruses behave like true negative strand RNA viruses. 

The arenaviruses life cycle starts with its attachment to the host cell. As for most viruses this early step is mediated by the interaction of a viral attachment protein to host cell-receptor(s). In the case of the prototypic LCMV, its broad cell tropism early suggested ubiquitous expression of conserved cellular receptor(s). In 1998, dystroglycan (DG) was identified as the first cellular receptor for LCMV and LASV [22] and subsequently for other OW arenaviruses and Clade C NW viruses [23]. In contrast, Clade B NW arenaviruses use transferrin receptor 1 (TfR1) as cellular receptor [24]. While pathogenic NW viruses like JUNV, MACV, GTOV, and SABV recognize human TfR1, non-pathogenic Clade B viruses use murine TfR1 orthologues [25]. The cellular receptors of Clade A NW arenaviruses remain currently unknown. The entry pathway of OW and Clade C NW arenaviruses is unusual, being independent of known regulatory proteins associated with endocytosis. LASV and LCMV infections are independent of clathrin, caveolin, dynamin, and actin, and dependent on lysobisphosphatidic acid (LBPA), an unusual phospholipid that is involved in the formation of intraluminal vesicles of the multivesicular body of the late endosome [26]. We speculate that the virus bound to the receptor gets internalized hijacking a pathway normally associated with endocytosis and degradation of α-DG. Productive infection with LASV and LCMV requires the endosomal sorting complex required for transport (ESCRT) components Hrs, Tsg101, Vps22, and Vps24, as well as the ESCRT-associated ATPase Vps4 and Alix [26]. This peculiar pathway may represent a strategy of the virus to avoid recognition by the innate immunity sensors [27]. A very different scenario is found in the case of the NW arenaviruses that use TfR1 for internalization. Similarly to α-DG, TfR1 localizes on the cellular surface of the target cells but, differently from α-DG, TfR1 possesses ligands which are known to be internalized by the receptor [28]. A strong Type I IFN response is usually associated with early stage of JUNV infection [29].

Upon receptor binding, mediated by the membrane-distal GP1, arenaviruses are taken up by endocytosis and delivered to acidified endosomes [30]. There, a pH-dependent membrane fusion step, mediated by the GP2 portion of arenavirus GP, results in the release of the viral ribonucleoprotein (RNP) into the cytoplasm of the infected cell, followed by viral RNA genome replication and gene transcription. Transcription by the viral L polymerase is initiated at promoters on each 5’end of the RNA segments, transcribing mRNA coding for the NP and L proteins. Transcription is terminated by structural motifs at the IGR downstream of the 3’end of each open reading frame [31]. Low levels of NP at the beginning of infection seem to prevent the virus polymerase from reading through the IGR, hence favoring transcription over replication. Subsequently, as NP accumulates, the viral polymerase shifts to a replicase mode and moves across the IGR, generating a full-length agRNAs which will serve as template for the synthesis of the GPC and Z mRNAs [32]. Complete viral genome is also amplified from the agRNA templates. Arenavirus infection induces discrete cytosolic structures for RNA replication [33]. 

The formation and release of arenavirus infectious progeny requires that assembled viral RNPs associate at the cell surface with membranes that are enriched in mature viral GP. Virion assembly and budding is orchestrated by the *bona fide* matrix protein Z involving late (L) domain motifs (PTAP and PPPY) [34,35,36]. Incorporation of arenavirus GPs into virion particles depends on the interaction of GP with Z [37], which recruits components of the endosomal sorting complex required for transport (ESCRT), in particular the ESCRT-I component Tsg101, that is crucial for virion budding [34,35]. 

## 2. The biosynthesis of the glycoprotein precursor

The envelope glycoprotein of Arenaviruses is synthesized as a polypeptide composed of an N-terminal stable signal peptide (SSP) and the SSP-containing GPC. Upon cleavage by cellular signal peptidases in the ER, GPC is further processed by the cellular proprotein convertase (PC) Subtilisin Kexin Isozyme-1 (SKI-1)/Site-1 Protease (S1P) into the GP1 and GP2 subunits [39,40,41] (Figure 1B). Post-translationally, the SSP acquires a myristoyl moiety at a Gly residue at position 2 [42] while the GP1/GP2 complex undergoes extensive N-glycosylation at multiple sites [43]. The interactions among the three subunits of the envelope glycoprotein, SSP, GP1, and GP2 are complex and not yet fully understood.

### 2.1. The signal peptide is not degraded and forms a stable tripartite complex with GP1/GP2

The role of the stable signal peptide (SSP) of arenaviruses has been comprehensively reviewed by a recent excellent review [44]. The arenavirus SSP has several unique characteristics in addition to its conventional function of targeting the nascent GPC polypeptide into the ER. Processing of nascent GPC by cellular signal peptidases occurs co-translationally and results in an unusually long (58 amino acids) signal peptide which does not undergo immediate degradation, but has a half-life of > 6h [45,46,47]. Standard signal peptides contain only one hydrophobic transmembrane domain but arenavirus SSP possess two hydrophobic regions separated by a hydrophilic loop containing a conserved positively charged Lys residue at position 33 (K33) [45]. Analysis of the JUNV SSP membrane topology revealed that both hydrophobic domains span the membrane with the N- and C-termini located in the cytosol and K33 facing the lumen/extracellular space [38]. SSP K33 is an important determinant for the pH-dependent fusion of the glycoprotein with the host cell membrane [48,49]. The SSP associates with the GP2 subunit, involving a Zn-binding motif [42,50,51,52,53]. Interestingly, recently discovered small molecule inhibitors of arenavirus cell entry target the molecular interface between SSP and GP2 [54], resulting in inhibition of pH-induced fusion. Lack of SSP myristoylation also affects fusion but not the formation of the SSP/GP1/GP2 complex [42]. The unusual arenavirus SSP is strictly required for proteolytic maturation of the GPC precursor [43,53]. Trans-complementation and interchanges among arenavirus GPC SSPs [55] are allowed but arenavirus SSP replacement with a generic signal peptide is not. In the case of JUNV GPC, the SSP was shown to be necessary for the GP to exit the ER by masking an ER-retention motif located on GP2 [53]. Lack of SSP would prevent the GP from being transported to the Golgi where SKI-1/S1P resides, thus averting maturation. 

### 2.2. Glycosylation of the GP1/GP2 complex

N-linked glycosylation is an essential process to help correct folding and intracellular transport of viral envelope GPs [56]. Analysis of the potential sites of N-glycosylation on arenavirus GPs revealed 4 conserved residues located on GP2 (with the exception of LCMV, DANV and LUJV), whereas the predicted sites on GP1 are multiple and show a lower degree conservation [43,57]. Recent studies showed that of the nine potential N-glycosylation sites of LCMV GPC, all the available sites were used on GP1 and two of three on GP2 [57]. All predicted N-glycosylation sites on LASV GPC (7 on GP1 and 4 on GP2) are used [43]. Mutation of some N-glycosylation sites prevented proteolytic processing, whereas others were dispensable for GPC cleavage. The role of N-glycosylation for GPC maturation and transport is complex and may be different according to the arenavirus species. Eichler *et al.* demonstrated that uncleaved LASV GPC present on the cell-surface remain EndoH sensitive, indicating the presence of mannose glycans, but absence of hybrid and complex N-glycans. Based on this result, cleavage of LASV GPC into GP1 and GP2 was suggested to be required for complex N-glycosylation upon exit from the ER [43]. Conversely, LCMV GPC cleavage was shown to occur after trimming of the mannose-rich N-glycans and their extension to complex N-linked sugars. 

### 2.3. GPC is cleaved by the cellular protease SKI-1/S1P

The majority of enveloped viruses shares a common mechanism of maturation based on the priming of their envelope GP precursors by host proteases in order to attain fusogenic properties [58,59,60,61,62,63]. Cleavage releases the fusion peptide, a hydrophobic stretch of 20-30 amino acids, which is able to act as an anchor in the target membrane, lowering the rupture tension of the lipid monolayer and promoting a negative curvature to ultimately allow joining the host-derived and virus membranes [64]. This strategy allows the envelope GP subunits to fold and oligomerize prior to assume, upon cleavage, a metastable conformation which undergoes drastic changes following receptor binding and/or drop of pH. The envelope precursor processing is also a mechanism to efficiently generate infectious particles since incorporation of mature envelope GPs is in general preferred over the uncleaved and fusion-incompetent ones [40,65,66]. Relative tissue abundance of the specific enzyme responsible for the processing contributes to determine the tropism of the enveloped virus whose growth may therefore be limited to specific cells/organs. 

**Figure 2:**
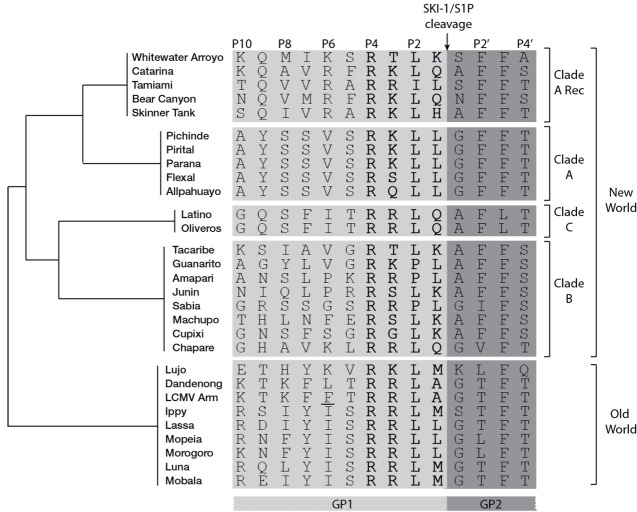
Amino acid sequence aligment of arenaviruses GPC residues surrounding the cleavage site (indicated by arrow). Residues of mature GP1 are in light gray while residues of mature GP2 are in darkgray. The four amino acids of clevage site motif are in bold. The schematic cladogram indicates the phylogenetic relationships between the viral clades is shown on the right. The LCMV cl13 strain amino acid variant F260L is underlined. Accession numbers are in supplementary table 1.

The arenavirus GPC is post-translationally cleaved at the highly conserved motif RX(hydrophobic)X↓, where X is any amino acid and hydrophobic is preferentially Leu (Figure 2) [67]. In the absence of GP1, GP2 spontaneously forms a trimer, in which each subunit is folded in a “hairpin”-like postfusion conformation of class I viral fusion proteins [68,69]. SKI-1/S1P is responsible for the cleavage of the GPCs of both OW [40] [39] and NW [41] arenaviruses. SKI-1/S1P belongs to the proprotein convertase (PC) family, which counts 9 members, so far, including 7 basic PCs (furin, PC1/3, PC2, PC4, PACE4, PC5/6, and PC7) and the self-inhibited proprotein convertase Subtilisin/Kexin type 9 (PCSK9). Differently from the other active members of PCs that cleave after clusters of (multi)basic motifs, the peculiar consensus sequence of SKI-1/S1P contains hydrophobic residues [70,71]. The protease is synthesized as an inactive zymogen that requires auto-processing of the N-terminal pro-domain at the B/B’ (RKVF↓RSLK↓) and C (RRLL↓) sites to attain full maturation [72]. In the cell, mature SKI-1/S1P localizes in the early Golgi, where the majority of its cellular substrates are cleaved, but also in the lysosomes and in the extracellular space, in a shed enzymatic form [71]. 

The biological activities of SKI-1/S1P are numerous and affect essential cellular functions. Among the best characterized SKI-1/S1P substrates are the Sterol Regulating Element Binding Proteins (SREBPs), transcription factors activated via limited proteolysis to control cell lipid homeostasis [72,73]. *In vivo* studies suggest that blocking SKI-1/S1P activity may be an alternative approach to lower plasma cholesterol [74]. The SREBPs activation process, common to other transcription factors, is known as regulated intramembrane proteolysis (RIP) and involves translocation to the Golgi compartment upon specific stimuli and subsequent proteolytic cleavage. Member of the CREB/ATF family of transcription factors including ATF6 [75] CREBH, Luman, CREB3L4, CREB4, and OASIS, have been identified as SKI-1/S1P substrates, related to the unfolded protein response (UPR) [76]. Very few cellular examples of non-transcription factor proteins are known to be cleaved by SKI-1/S1P. Examples are N-acetylglucosamine-1-phosphotransferase, a key enzyme for the sorting of proteins into the lysosomal compartment [77], pro-brain derived neurotrophic factor (BDNF) [78] and repulsive guidance molecule (RGMa) [79]. SKI-1/S1P cleaves proBDNF within its pro-domain but the biological significance of this processing is not known. Similarly, RGMa processing by SKI-1/S1P seems to be required although the cleavage sites have not yet been identified. The protease was also found to be crucial for normal bone and cartilage formation [80,81], and mice coat pigmentation [82], but the substrates involved in these biological processes are still to be discovered. In mice, SKI-1/S1P K.O. shows a lethal phenotype with the embryos dying at an early developmental stage [83]. Even incomplete inhibition of SKI-1/S1P causes severe effects: homozygosity for the *woodrat* mutation, which shows partial loss of enzyme function, leads to hypersensitivity to dextran sodium sulfate-induced colitis [84], and causes enhanced embryonic mortality [85].

In contrast to cellular substrates of SKI-1/S1P that are processed in the median Golgi, LASV GPC is cleaved in the ER/cis-Golgi and LCMV GPC in a late Golgi compartment [39,71,86]. The way the virus is able to hijack active SKI-1/S1P in these specific subcellular compartments is not known. However, switching the LCMV GPC RRLA↓ motif into RRLL↓ present in LASV GPC re-direct the cleavage of the latter from a late Golgi compartment to the ER/cis-Golgi. In contrast, a LASV GPC chimera carrying the LCMV “RRLA↓” motif, is not cleaved despite following normal sorting to the plasma membrane [86]. Thus, available evidence suggests that the specific amino acid sequence at the cleavage site may contribute to the processing at specific sub-cellular localizations. No clear information is currently available about the maturation of other arenavirus GPCs, although some evidences suggest JUNV GPC processing in the Golgi [87]. 

A characteristic common to most arenavirus GPCs [67] (Figure 2) is the striking resemblance of their processing sites to SKI-1/S1P auto-cleavage motifs: while African OW and NW clade B arenavirus GPCs mimic the C-auto-processing site “RRLL↓”, NW arenavirus GPCs resemble the B-auto-processing site “RSLK↓”. The mimicry indicates convergent evolution towards a common maturation mechanism resembling SKI-1/S1P auto-processing, which is not evident in other cellular SKI-1/S1P substrates. The advantage may account for the virus’ ability to avoid interference with cellular functions in order to persist in the reservoir rodent host. Accordingly, the SKI-1/S1P-dependent activation of ATF6 is normally induced by LCMV GPC during acute infection. As soon as GPC expression is down-regulated during the transition from acute to persistent infection [88], the UPR response triggered by ATF6 disappears [89]. However, the reason behind the selective association “RRLL↓”-OW arenavirus and “RSLK↓”-NW arenavirus are currently unclear.

Specific GPC residues surrounding the cleavage site were shown to influence maturation. An F259A point mutation at P7 position of LCMV GPC greatly impairs processing [39]. Analysis of peptides mimicking the cleavage site of LASV GPC revealed that cleavage efficiency may be negatively affected by the presence of a non-aromatic residue at P7 position, indicating the presence of an unusually large catalytic pocket able to accommodate residues distal from the actual processing site [70]. Indeed, in comparative studies, the YISRRLL↓ sequence mimicking the cleavage site of LASV GPC is the best substrate to assess *in vitro *SKI-1/S1P enzymatic activity [77,78,90]. 

The N-terminus of LASV GP2 contains two stretches of highly conserved hydrophobic amino acids. Alanine scanning of the N-terminal 260-266 and 276-298 sequences revealed that maturation of the envelope GPC does not occur in presence of single point mutations at positions 260, 261, 262, 280, 284, 285, 286, 292, and 293, despite GPC folding and reaching the plasma membrane [91]. Indeed, GPC cleavage is not essential for transport to the plasma membrane, yet only the fully mature complex is found on budding particles [66,72,74]. A similar effect is observed in the case of the LASV GPC N-glycosylation mutants S367A and S375A, which are transported normally to the cell surface but fail to be cleaved [43]. LASV GP1 glycosylation at position 81, 91, 101, and 121 are required for proteolytic processing but not cell surface localization. In contrast, mutations that abolish normal glycosylation at positions 111, 169, and 226 are not crucial for the precursor maturation [43]. The data at hand thus suggest that SKI-1/S1P-mediated processing may require specific tertiary/quaternary structures of the GP1/GP2 complex that are disrupted by specific point mutations deletions. 

A large body of evidence indicate a crucial role of the cytoplasmic domains of viral envelope GPs in assembly, fusogenicity, and infectivity of enveloped viruses, including Newcastle disease virus [92] Measles virus [93], and HIV-1 [94]. Similar observations have been made with arenaviruses. Removal of the entire LCMV GP2 cytosolic tail as well as partial deletion of the three C-terminal amino acids RRK prevent maturation of GPC without interfering with its normal transport to the cell surface [65]. As similar role of amino acid residues in the cytoplasmic domain for GPC cleavage was observed by Schlie et al. in the context of LASV GPC [95]. In arenaviruses, lack of cleavage has thus a drastic impact on infectious particles production since the process of GP incorporation into nascent virions discriminates processed over unprocessed GPC.

### 2.4. Targeting GPC maturation is a promising strategy against arenavirus infection

Similarly to other enveloped viruses, whose GPs are processed by PCs, such as HIV-1 and influenza virus, arenaviruses strictly rely on SKI-1/S1P for productive infection. In SKI-1/S1P-deficient cells, uncleaved GPCs are correctly transported to the plasma membrane but fail to be incorporated into budding particles, which remain “naked”, and lack infectivity [40,51,65]. Thus, GPC maturation represents an attractive drug target for complementary therapeutic intervention in addition to ribavirin. Over the past ten years, several SKI-1/S1P inhibitors have been synthesized and identified [67,70,96], but only few showed potential applications based on their cell-permeability, cytotoxicity and specificity. We [97] and others have shown that peptide- (dec-RRLL-cmk) or protein-(PDX-variants) [98] based SKI-1/S1P inhibitors efficiently blocks arenavirus cell-to-cell propagation. The on-target effect of dec-RRLL-cmk was demonstrated by using a recombinant LCMV bearing a furin-cleavable GPC, which was only slightly affected by the treatment [97]. More importantly, these investigations provided proof-of-concept that inhibition of GPC processing can contain the infection. Unfortunately, the use of the small molecule dec-RRLL-cmk was restricted due to its toxicity and follow-up studies *in vivo* were not feasible [99]. Rational design of SKI-1/S1P inhibitors is limited by the lack of structural information about the enzyme. As an alternative approach, Pfizer Inc. performed high-throughput small molecule screening using a homogeneous enzyme activity assay format based on purified soluble SKI-1/S1P that lead to the discovery of the aminopyrrolidineamide-based inhibitor PF-429242 with an *in vitro* IC_50_ in the nanomolar range [100]. The compound was potent in blocking SREBP-2 activation *in vivo*, significantly lowering plasma cholesterol levels [74]. PF-429242 is a potent inhibitor of cell-to-cell propagation of OW arenaviruses *via* direct interference with the SKI-1/S1P-mediated GPC cleavage [101]. The on-target effect of PF-429242 was confirmed *in vitro* on synthetic peptides mimicking the cleavage site of the LASV GPC: processing of the LASV GPC derived Ac-IYISRRLL-MCA peptide is inhibited by PF-429242 with an IC_50_ of 130 nM [99]. The small drug molecule appears to be effective against NW arenaviruses as well [99]. Combinatorial PF-429242 treatment with the well known broad anti-viral ribavirin shows a synergetic effect *in vitro* [99], suggesting that the administration of a SKI-1/S1P inhibitor may help to combat the infection. 

A hallmark of persistent infection is the down-regulation of GPC expression to almost undetectable levels with respect to the viral NP [102]. During this phase, infectious particles are still produced. It is conceivable that any perturbation of the GPC maturation may have drastic effects, as the envelope GP becomes the limiting factor for virus production and its inhibition cannot be compensated easily. Accordingly, in LCMV persistently infected cells the virus is rapidly cleared by PF-429242 treatment with apparently no emergence of escape variants [99]. Early studies in SKI-1/S1P-deficient cells showed that LCMV and JUNV can persist over long periods with a low probability of emergence of SKI-1/S1P independency before extinction [97]. In terms of drug development, this is of great importance because it suggests that the emergence of SKI-1/S1P-independent arenaviral variants is a rare event, further supporting the potential of SKI-1/S1P inhibitors as an anti-viral strategy. It is currently unclear why arenaviruses cannot escape SKI-1/S1P deficiency. Possibly, SKI-1/S1P-mediated GPC cleavage is crucial not only for the liberation of the N-terminal GP2 fusion peptide. Interestingly, addition of exogenous SKI-1/S1P is incapable of maturating GPC expressed at the surface of SKI-1/S1P null cells, suggesting that processing must occur in a specific sub-cellular compartment [86]. The relevance for SKI-1/S1P for productive arenavirus infection *in vivo* is further illustrated in mice homozygous for the hypomorphic *Mbtps1* wrt allele (woodrat mutation), carrying a defective but still partially active SKI-1/S1P are resistant to LCMV infection [85]. 

Given the importance of arenavirus envelope GPC processing for productive infection and its dependency on a protease involved in several crucial host functions, efforts have been made to understand how viral substrate and the enzyme interact, with the goal to eventually develop substrate-specific drugs. Biochemical characterization of SKI-1/S1P-GPC interaction revealed that auto-processing at the B/B’ sites of SKI-1/S1P is crucial for effective GPC maturation but not cellular SREBP-2 activation [86]. The exact reasons for the specific requirement of B/B’ autoprocessing for efficient cleavage of viral, but not cellular substrates are currently not known. However, this remarkable difference suggests that inhibitors of SKI-1/S1P autoprocessing may show some specificity for viral substrates with less of an effect on processing of cellular substrates. Moreover, the data at hand indicate that viral and cellular substrates of SKI-1/S1P are processed in distinct subcellular compartments. In uninfected cells, membrane-associated SKI-1/S1P is found predominantly in the early Golgi where cellular SKI-1/S1P substrates are cleaved [103]. In contrast, LASV GPC is cleaved early in the secretory pathway and LCMV GPC was shown to be processed in a late Golgi or post-Golgi compartment [86]. The non-overlapping sub-cellular localization of viral and cellular substrates has been further supported by the finding that SKI-1/S1P mediated processing of ATF-6 is unaffected upon infection [89]. Targeting the protease in specific sub-cellular compartments may thus affect predominantly processing of viral GPCs. In this context, the recent reported protein inhibitor SKI-1/S1P Spn4A.RRLL(r), a modified serpin with SKI-1/S1P specificity, coupled to HDEL ER-retention sequence [104] may act as a LASV specific inhibitor. Accordingly, Spn4A.RRLL(r) does inhibit SKI-1/S1P *in vitro* without affecting the SREBP-2 pathway. As mentioned above, residues surrounding GPC processing site affect substrate-enzyme recognition. In particular, an aromatic amino acid at P7 was found to be crucial for processing of both LASV [70] and LCMV GPC [39], making this region of the GPC an attractive anti-viral drug target given that the interference with P7-S7 interaction should not perturb the catalytic triad and thus general enzymatic activity.

The envelope glycoprotein processing is a key step in arenavirus life-cycle. *In vitro* inhibition of SKI-1/S1P-mediated processing of GPC greatly limits virus cell-to-cell propagation, thus representing a promising antiviral strategy. *In vivo* evaluation of SKI-1/S1P inhibitors as antivirals, e.g. PF-429242, would be of great importance to understand the efficacy of interfering with normal GPC maturation on arenavirus infections. During drug treatment, dietary supplementation of cholesterol and other lipids may be taken into account to compensate for plasma cholesterol reduction, resulting from SREBPs inhibition. Novel substrate specific SKI-1/S1P inhibitors may be of great value to minimize side effects. 

## 3. Role of the envelope glycoprotein on virus assembly and particle formation

Viral infection induces a global reprogramming of cellular processes, including alterations the organization of intracellular membranes and organelles [105,106]. Assembly of the viral components, and in particular GP, to form progeny infectious particle is still a process that remains for many aspects unknown. As introduced above, the driving force for arenaviruses budding is the matrix protein Z [34,35] which interacts *via* its late or RING domain with Tsg101, a member of the ESCRT machinery [34,36]. Myristoylation of Z is crucial for the correct matrix protein localization and therefore budding [107]. Z possesses *per se* budding properties and does not require any other viral protein to accomplish its function. However, the ability to produce infectious particles resides on the assembly of the four different viral proteins in a synchronized fashion. Thus, Z is required to interact with the SSP/GP1/GP2 complex that decorates the surface of the nascent particle. Confocal and biochemical analyses showed a direct LCMV and LASV GP1/GP2-Z interaction, dependent on the stable signal peptide of the GP complex [37]. Cells that express LASV GPC together with Z show a partial co-localization in vesicle-like structures near the nucleus [108]. Sub-cellular localization of the envelope glycoprotein is not modified by the presence of Z [34]. Z myristoylation, but not on the Z late (L) or RING domain was shown to be responsible for the matrix protein binding to the envelope GP in OW arenaviruses [37]. However, disrupting the Z RING domain resulted in a drastic loss of TCRV and JUNV GP incorporation, suggesting some differences in the detailed molecular interactions underlying virion assembly in OW and NW viruses [109]. Further analyses are required to better understand the role of the GP complex in the virus budding events. The mechanisms underlying the selective incorporation of cleaved over uncleaved envelope GPs remain unclear and may involve different tertiary/quaternary structures of the mature SSP/GP1/GP2 complex in comparison to immature GPC [20]. Inhibition of virus assembly and budding is becoming more and more attractive as a drug target, opening the possibility to block egress of progeny virions from already infected cells. A detailed analysis of the molecular interactions underlying the recruitment of processed GP, Z, and the RNP into budding zones in infected cells will likely reveal promising drug targets for novel therapeutics to combat human arenavirus infection. 
